# *Salmonella* T3SS effector SseK1 arginine-glycosylates the two-component response regulator OmpR to alter bile salt resistance

**DOI:** 10.1038/s41598-023-36057-9

**Published:** 2023-06-03

**Authors:** Md Kamrul Hasan, Nichollas E. Scott, Michael P. Hays, Philip R. Hardwidge, Samir El Qaidi

**Affiliations:** 1 College of Veterinary Medicine, Kansas State University, Manhattan, KS 66506 USA; 2 Department of Microbiology and Immunology, University of Melbourne Within the Peter Doherty Institute for Infection and Immunity, Melbourne, 3000 Australia; 3 Boehringer-Ingelheim Vetmedica, Ames, IA 50010 USA

**Keywords:** Microbiology, Bacteriology, Clinical microbiology

## Abstract

Type III secretion system (T3SS) effector proteins are primarily recognized for binding host proteins to subvert host immune response during infection. Besides their known host target proteins, several T3SS effectors also interact with endogenous bacterial proteins. Here we demonstrate that the *Salmonella* T3SS effector glycosyltransferase SseK1 glycosylates the bacterial two-component response regulator OmpR on two arginine residues, R15 and R122. Arg-glycosylation of OmpR results in reduced expression of *ompF*, a major outer membrane porin gene. Glycosylated OmpR has reduced affinity to the *ompF* promoter region, as compared to the unglycosylated form of OmpR. Additionally, the *Salmonella ΔsseK1* mutant strain had higher bile salt resistance and increased capacity to form biofilms, as compared to WT *Salmonella*, thus linking OmpR glycosylation to several important aspects of bacterial physiology.

## Introduction

*Salmonella* is responsible for ~ 1.35 million infections in the USA each year^1^. Identifying virulence factor mechanisms involved in pathogenesis and environmental persistence is essential to finding better approaches to reduce *Salmonella* disease burden. *Salmonella* uses a specialized secretion system named Type Three Secretion System (T3SS) to inject effector proteins into host cells^2–4^. Many of these effector proteins inhibit host immune responses. The *Salmonella* T3S effector SseK and its ortholog NleB target host cell immune response pathways to reduce host inflammatory responses^5^. SseK and NleB are glycosyltransferases that glycosylates several host proteins and on specific arginine residues and interfere with their physiological function^6–8^. For example, SseK1 glycosylates the death domain containing protein TRADD (Tumor necrosis factor Receptor type 1-Associated DEATH Domain protein), and Tubulin Folding Cofactor TBCB^7,9–11^. SseK2 glycosylates FADD (FAS-Associated Death Domain protein)^7^. NleB from EHEC is known to target FADD, TRADD, RIPK1 (Receptor Interacting Protein Kinase 1), TNFR1 (Tumor Necrosis Factor Receptor superfamily 1), and HIF-1α protein^12,13^. These modifications ultimately interfere with proper physiological functions of the target proteins. Besides death domain containing proteins, NleB1 glycosylates arginine residues of GAPDH^13^. Arginine glycosylation of GAPDH prevents its interaction with TRAF2 and the subsequent induction of NF-kB signaling. Deletion of any one of these glycosyltransferase effectors is correlated with reduced bacterial virulence in a mouse model^8,9^.

In addition to their known host targets, our group and others have recently demonstrated that NleB/SseK orthologs also glycosylate bacterial proteins. For example, *Citrobacter rodentium* effector NleB Arg-glycosylates the glutathione synthase GshB, leading to enhanced glutathione synthase activity and consequently increased resistance to oxidative stress^14^. *Salmonella* T3SS effector SseK1 also plays a significant role in methylglyoxal detoxification by glycosylating the GloA, GloB, GloC, and YajL proteins in this pathway^15^. Additionally, our latest study on SseK1 intrabacterial activity demonstrates that SseK1 upregulates UDP-GlcNAc synthesis by glycosylating NagC and GlmR^16^.

Two-component response regulators are used by bacteria to sense and respond accordingly to the surrounding environment^17–19^. Two-component systems are comprised of a membrane-bound kinase and a corresponding response regulator that exerts the effect of the external stimuli typically by regulating transcription of target genes^20^. It was recently described that SseK3-mediated Arg-glycosylation plays an important role in modulating the DNA-binding activity of *Salmonella* PhoP, a two-component response regulator^21^. Another critical two component response regulator of *Salmonella* is the EnvZ-OmpR system. The EnvZ-OmpR system is known for its regulatory effects on the major outer membrane porins OmpF and OmpC in response to extracellular pH and osmolarity change^22–24^. With increasing osmolarity, EnvZ phosphorylates the response regulator protein OmpR. Once phosphorylated, the binding affinity of OmpR to target gene promoters increases, resulting in their transcriptional upregulation^25,26^. Additionally, OmpR can also non-canonically regulate transcription of its target genes under its non-phosphorylated state^23^. Besides regulating several stress related genes^27–32^, OmpR also regulates the expression of effector genes in *Salmonella* Pathogenicity Island 2 (SPI2) which is especially relevant to the intracellular adaptation of *Salmonella*^26,33–37^.

In a previous study, pull down experiments combined with mass spectrometry sugar analysis were performed to identify novel host targets of effector glycosyltransferase, wherein OmpR was unexpectedly detected as SseK1 target. Here we found that OmpR is glycosylated by SseK1. Glycosylation of OmpR leads to decreased expression of its target gene *ompF*, presumably by reducing the binding affinity of OmpR to the *ompF* promoter region. We also found that whereas a *Salmonella ΔompR* mutant has a significant growth defect in the presence of bile salts and a reduced capacity to form biofilms, a *Salmonella ΔsseK1* mutant has the opposite phenotype, indicating an overall repression of OmpR transcriptional activity through SseK1 mediated-glycosylation.

## Results

### SseK1 glycosylates OmpR

**Figure 1:**
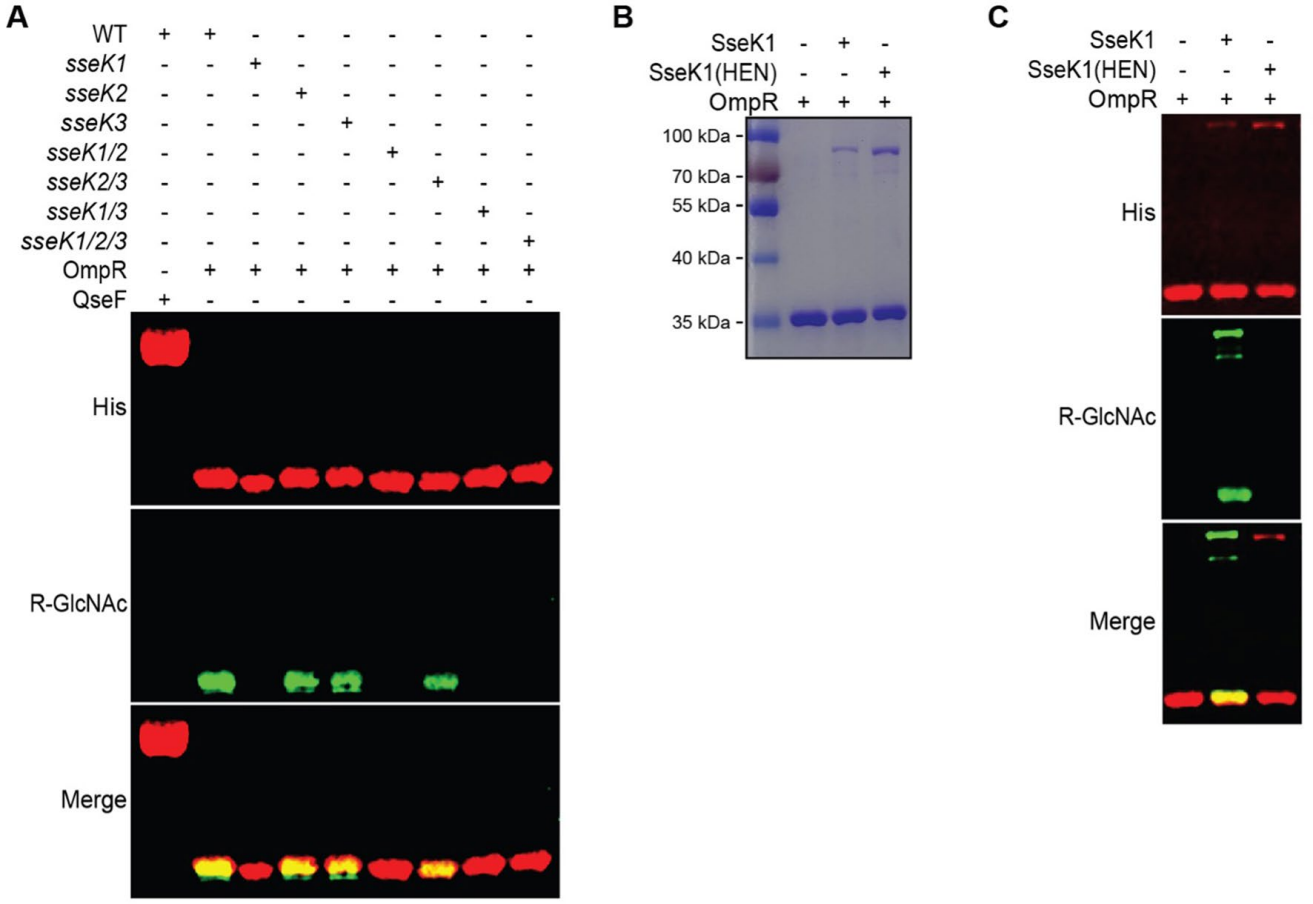
SseK1 Arg-glycosylates OmpR. (**A**) Western blot analysis of intra-bacterial glycosylation of OmpR and QseF in different *Salmonella sseK* mutant strains; (**B**) SDS-PAGE image of the enzymes and substrates used for in vitro glycosylation assays; (**C**) Western blot analysis of in vitro glycosylation of OmpR in the presence of active or inactive (HEN) forms of SseK1.

*Salmonella enterica* serover *Typhimurium* encodes three SseK glycosyltransferase paralogs named SseK1,SseK2, and SseK3. We expressed OmpR in wild type, single, double, and triple *sseK* mutants. We used an anti R-GlcNac monoclonal antibody to conduct western blot analysis of cell lysates expressing recombinant His tagged OmpR to investigate which SseK paralog glycosylates OmpR. Only SseK1 glycosylated OmpR (Fig. 1A). Additionally, another two component response regulator, QseF, was not glycosylated by any of the SseK paraologs, serving as a negative control for the assay (Fig. 1A). We conducted an in vitro glycosylation assay with purified SseK1, a catalytically inactive mutant of SseK1 (SseK1 HEN mutant), and OmpR (Fig. 1B). In vitro glycosylation assays demonstrated that OmpR is glycosylated by wild-type SseK1 whereas SseK1 HEN failed to glycosylate OmpR with GlcNAc (Fig. 1C).

**Figure 2:**
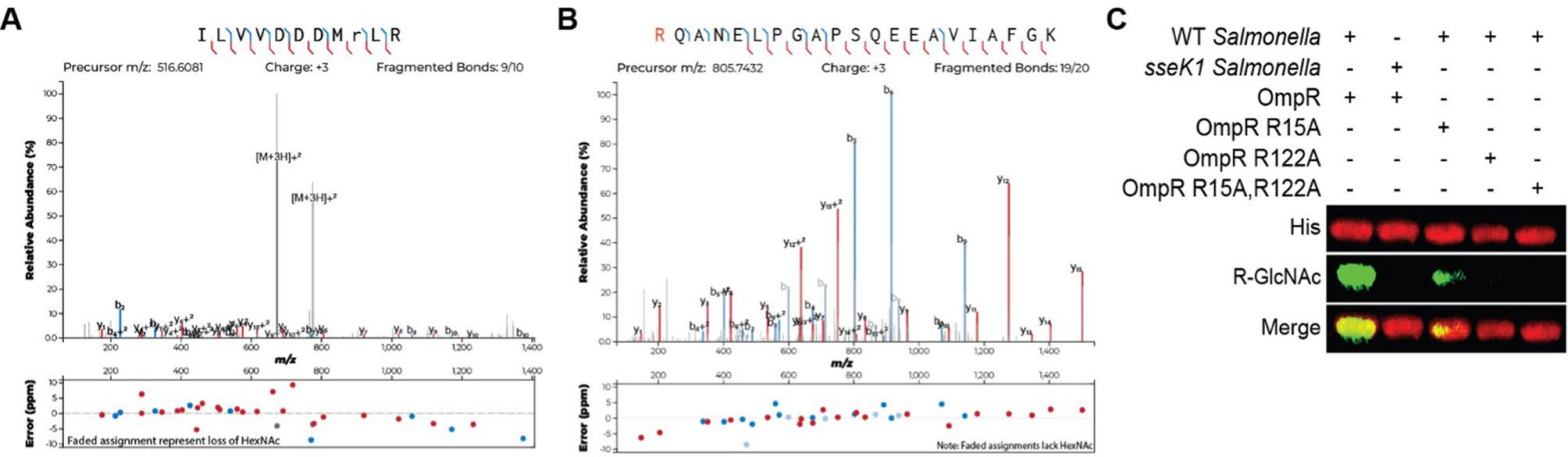
Identification of OmpR glycosylation sites. (**A**) HCD spectra of the in vivo glycosylated OmpR tryptic peptides containing glycosylated R15; (**B**) R122; (**C**) Western blot verification of Arg-glycosylation of WT and R-to-A point mutations of OmpR.

After confirming that OmpR is a bacterial SseK1 target, we wanted to identify the specific OmpR glycosylation sites. We detected, using mass spectrometry suger analysis, two potential OmpR arginine residues, R15 and R122 (Fig. 2A,B). We validated the mass spectrometry data by creating recombinant OmpR proteins with R15A, R122A, and R15,R122A point mutations. We expressed the recombinant OmpR point mutants in WT *Salmonella*. Western blot analysis of cell lysates indicated that OmpR R12A is glycosylated, albeit at a reduced level. The R122A and R15,R122A point mutants were not glycosylated, indicating that R122 is the primary SseK1 target residue (Fig. 2C).

### Glycosylation of OmpR results in altered expression of ompF

**Figure 3:**
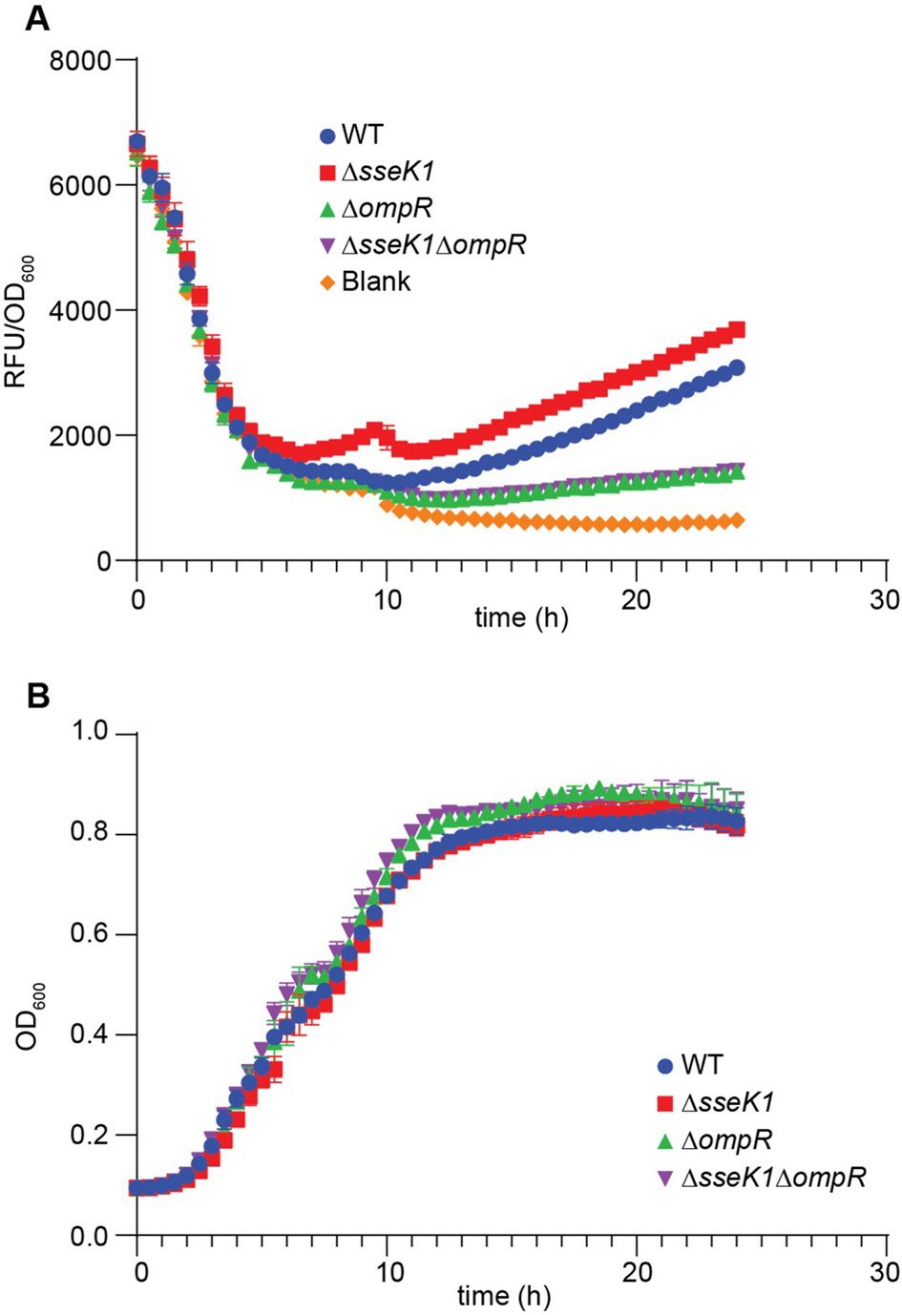
*ompF::mrfp* transcriptional reporter assay. (**A**) Measurement of mRFP expression levels of *ompF::rfp* transcriptional fusions in WT *Salmonella enterica* and its *ΔsseK1* or *ΔompR* or *ΔsseK1/ΔompR* derivatives. mRFP levels are expressed as RFU (relative fluorescence units)/OD_600_ ratio; (**B**) Growth rates of *Salmonella* strains used in the mRFP fusion assay.

OmpR is a transcriptional regulator. To understand the consequence of OmpR glycosylation by SseK1, we measured the transcription of an important OmpR-regulated gene, *ompF*. OmpF is a key outer membrane porin protein that is essential for *Salmonella* adaptability to pH and osmolarity stress^30,38,39^. We used a mRFP (monomeric red fluorescent protein) transcriptional fusion assay wherein the mRFP gene was fused with the upstream promoter region of *ompF* and the RFP levels were measured in either WT, *ΔsseK1, ΔompR*, or *ΔsseK1/ompR* double mutant strains. Our mRFP transcriptional reporter assay data showed a decreased mRFP signal for *ompF* promoter fusions in WT *Salmonella* as compared to the *ΔsseK1* strain (Fig. 3A). As expected, *ΔompR* and the *ΔsseK1/ompR* double mutant showed significantly reduced activity of the *ompF* promoter (Fig. 3A). There was no significant growth difference among the strains (Fig. 3B).

**Figure 4:**
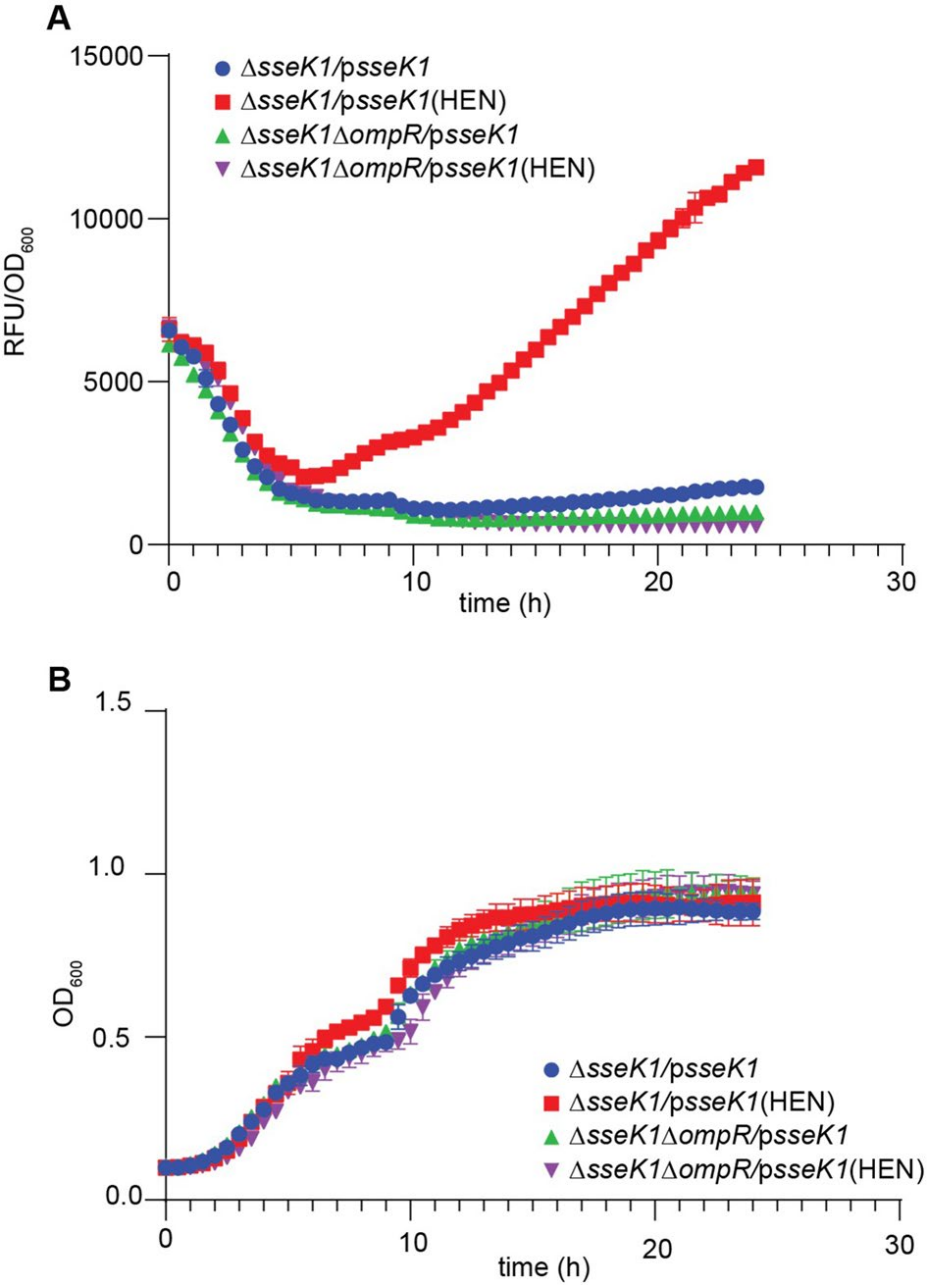
*ompF::mrfp* transcriptional reporter assay of *ΔsseK1* complemented strains. (**A**) Measurement of mRFP expression levels of *ompF::rfp* transcriptional fusions in *ΔsseK1* or *ΔsseK1/ompR Salmonella* complemented with either *sseK1* or *sseK1* HEN. mRFP levels are expressed as RFU (relative fluorescence units)/OD_600_ ratio; (**B**) Growth rates of *Salmonella* strains used in the mRFP fusion assay.

To further validate our findings, we complemented *ΔsseK1 Salmonella* with either *sseK1* or *sseK1* HEN (inactive) and repeated the mRFP transcriptional assay. Our assay showed a significant reduction of *ompF* promoter activity in the SseK1-complemented strain, as compared to the SseK1 HEN (inactive)-complemented strain (Fig. 4A). As expected, *Salmonella ΔompR* and *ΔsseK1/ompR* mutant strains failed to induce any significant mRFP signal (Figs. 3A and 4A). There was no significant growth difference among the strains (Fig. 4B).

**Figure 5:**
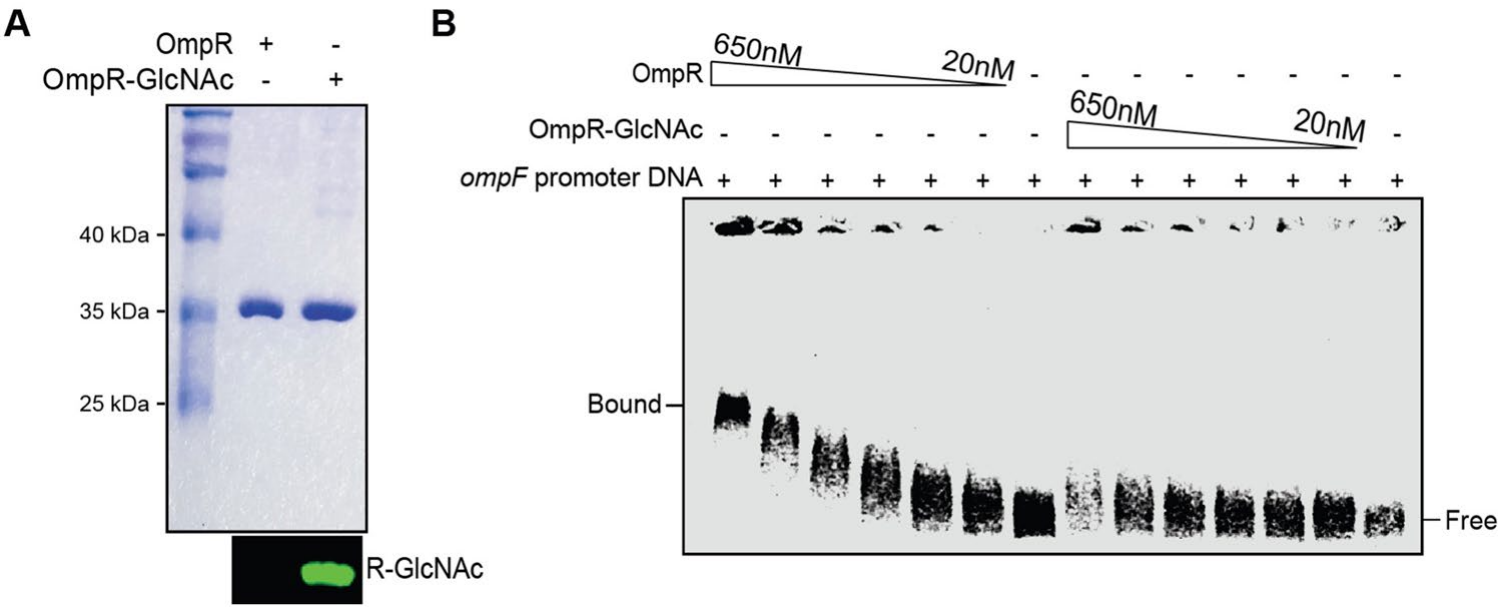
Glycosylation reduces OmpR DNA binding affinity. (**A**) Purification of native and Arg-glycosylated OmpR combined with validation of glycosylation by Western blotting; (**B**) EMSAs comparing the DNA-binding activity of OmpR and OmpR-GlcNAc towards *ompF* promoter DNA.

To understand the molecular mechanism of SseK1-mediated reduced promoter activity of OmpR target genes, we conducted an EMSA assay in which purified native or Arg-glycosylated OmpR proteins (Fig. 5A) were incubated with fluorescently labelled *ompF* promoter region DNA*.* We observed reduced affinity of glycosylated OmpR to its target DNA as compared to unglycosylated OmpR (Fig. 5B), consistent with the *ompF* promoter activity assay data.

### *Salmonella ΔsseK1* strain has increased bile tolerance and biofilm formation capacity

**Figure 6:**
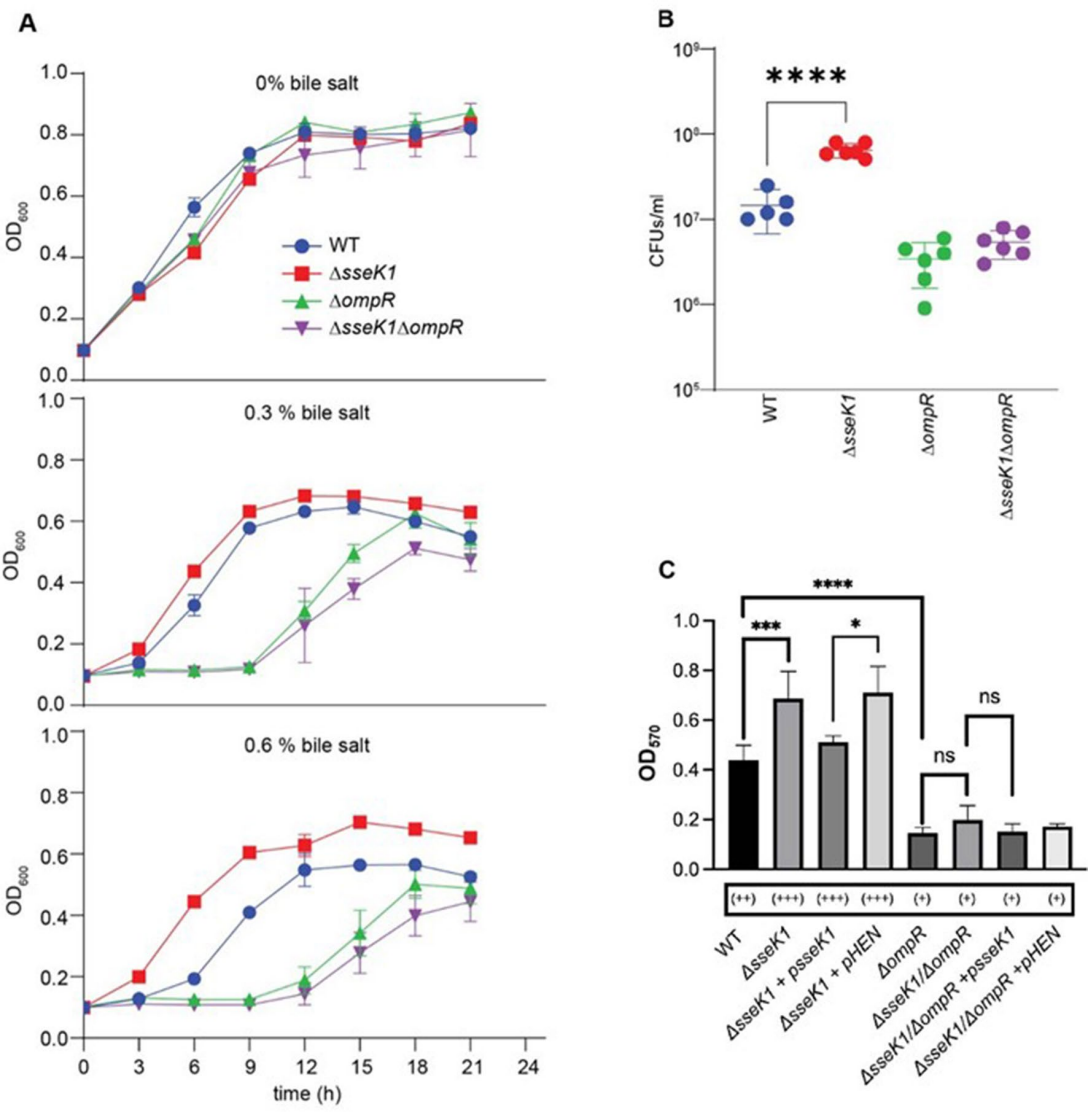
OmpR glycosylation results in altered bile salt tolerance and biofilm formation capacity of *Salmonella*. (**A**) Growth curve analysis of WT *Salmonella enterica* and its *ΔsseK1* or *ΔompR* or *ΔsseK1/ompR* derivatives in no bile salt, 0.3% bile salt (sodium deoxycholate), 0.6% bile salt; (**B**) CFU counts of *Salmonella* strains after 16 h growth in 0.6% bile salts in LB; (**C**) Biofilm formation capacity of different *Salmonella enterica* mutant and complemented strains. Mutant strains contain empty vector. Complemented strains harbors plasmids expressing either WT *sseK1* or *sseK1* HEN point mutant. Biofilms were also classified as described by Christensen et al.^73^ into the following categories: non-adherent (0), weakly (+), moderately (++), or strongly (+++) adherent, based upon the ODs of bacterial biofilm.

OmpR modulates several critical *Salmonella* pathways^34,35,40,41^, one of which is bile salt tolerance^38^. Bile salts are natural antimicrobial compounds produced by the host to reduce pathogen proliferation^42^. OmpR is a positive regulator of bile salt tolerance^43^ and thus we wanted to investigate whether glycosylation of OmpR by SseK1 has any effect on bile salt tolerance. We measured the growth of WT, *ΔsseK1*, *ΔompR*, and *ΔsseK1/ompR* double mutant *Salmonella* strains for their capacity to grow in the presence of bile salts. Our data shows that in the presence of 0.6% sodium deoxycholate, *ΔsseK1 Salmonella* strains grew faster than WT *Salmonella* (Fig. 6A,B). In the presence of 0.3% sodium deoxycholate, the difference in growth was reduced (Fig. 6A). In contrast, the *Salmonella ΔompR* mutant grew poorly in bile salts, as expected (Fig. 6B). The *ΔsseK1/ompR* double mutant also grew poorly in the presence of bile salts, indicating that the observed increased bile salt tolerance of *ΔsseK1* mutant *Salmonella* is exerted through OmpR (Fig. 6B). No significant growth difference between these strains was observed when they were grown in the absence of sodium deoxycholate (Fig. 6A).

Another key virulence aspect of *Salmonella* is the biofilm formation process^44–46^. OmpR is a positive regulator of biofilm formation in several pathogens including *E. coli*, *Klebsiella*, and *Salmonella*^43,47,48^. We hypothesized that OmpR glycosylation might have an impact on *Salmonella* biofilm formation capacity. We compared the biofilm formation ability of *Salmonella* WT, *ΔsseK1*, *ΔompR* and *ΔsseK1/ompR*. *ΔsseK1* produced significantly more biofilm as compared to WT *Salmonella* (Fig. 6C). As expected, the *ΔompR* strain formed significantly less biofilm as compared to the WT strain (Fig. 6C). Additionally, we observed no statistically significant difference in biofilm production between *ΔompR* and the *ΔompR/sseK1* double mutant, indicating an epistatic effect of the *ompR* mutation on *sseK1* for this particular phenotype (Fig. 6C). This observation was further supported by the complementation studies where expression of *sseK1* did not complement the biofilm formation defect seen in *ΔompR/sseK1* double mutant (Fig. 6C).

## Discussion

*Salmonella* harbors two Pathogenicity Islands—SPI1 and SPI2. Although encoded by a gene located outside SPI1 and SPI2, SseK1 was shown to be secreted by both T3SS1 and T3SS2 secretion systems^49^. Once translocated, SseK1 glycosylates host proteins to reduce host immune responses during infection^10^. SseK1 and its ortholog NleB also glycosylate several bacterial proteins. Here we demonstrate that among the SseK paralogs, only SseK1 targets the two-component response regulator OmpR indicating a highly specific interaction, the effect of which was observed through the differential expression of an OmpR target gene—*ompF*.

The regulation of *ompF* by OmpR is a complex multiunit affair with several other transcription factors working in concert to either up- or down-regulate *ompF* expression^24,50^. Although the total amount of outer membrane porins (OMPs) of a cell generally does not change significantly, the combination of different OMPs is altered in response to different environmental stimuli. These changes are orchestrated largely by the ratio of phosphorylated and unphosphorylated OmpR along with several other regulators of OMP expression^48,51–53^. While conducting the first set of mRFP fusion assays we used either WT or mutant *Salmonella* strains to assess the effect of endogenously expressed *sseK1* on OmpR transcriptional activity. A significant differential expression level of *ompF* promoter activity was detected under these conditions, indicating that OmpR glycosylation by SseK1 is likely to occur as an evolutionary bacterial adaptation to specific stress conditions.

Since *ompF* is part of the OmpR regulon^54^, we investigated whether OmpR glycosylation alters its DNA binding affinity of *ompF* promoter. Our results showed a reduction in DNA binding capacity of glycosylated OmpR as compared to the unglycosylated OmpR. Interestingly, the OmpR glycosylation sites detected by mass spectrometry analysis and validated by site directed mutagenesis are located outside the OmpR DNA-binding motif^55,56^, rather than in the N-terminal response regulatory region. Moreover, the OmpR glycosylation sites R15 and R122 are not in vicinity of the D55 phosphorylation target site^22,51,57^. One potential explanation of these data is that glycosylation could alter the conformation of the OmpR DNA binding domain to prevent its binding to target promoters. The silenced affinity of OmpR to its target promoters upon glycosylation by SseK1 possibly evolved as a gene modulatory mechanism to reverse the enhanced affinity of this transcriptional factor to DNA upon phosphorylation by EnvZ at the residue D55. Thus, this study illustrates an original example of bacterial transcription gene regulation where the same transcription factor can undergo two different post-transcriptional modifications (phosphorylation and glycosylation) with opposite effects on target gene expression.

Two-component response regulators are key players in bacterial adaptation to dynamic environmental conditions^18,19,58^. OmpR is vital for *Salmonella* adaptation in changing environments. As such, we investigated the significance of OmpR glycosylation by SseK1. Even though our finding that *Salmonella ΔsseK1* strain has increased bile salt and biofilm production capacity seems counterintuitive at the first glance, we believe it can be rationally explained. Our experiments were performed in laboratory conditions with rich media which are not always ideally representative of the natural pathogen lifestyle. In *Salmonella*, SseK1 is transcribed from SPI-2, which is upregulated during its intracellular lifestyle^4,33–35,59–61^. While inside the host cell, increased SseK1 expression could lead to increased glycosylation of OmpR, leading to downregulation of pathways that are of less significance in the intracellular environment, such as bile salt tolerance and biofilm formation. Conversely, outside of host cells, SPI-2 is not induced^62–65^, leading to less SseK1 which then leads to lower inhibition of OmpR activity. This would allow WT *Salmonella* to exhibit more of a *ΔsseK1* mutant-like phenotype during its extracellular lifestyle, with increased bile salt tolerance and surface adherence.

In contrast to our recent work on NagC glycosylation leading to increased DNA binding affinity^16^, here we observed decreased DNA binding affinity of glycosylated OmpR. One significant difference between glycosylated NagC and glycosylated OmpR is that the glycosylated Arg residues of NagC reside within the HTH DNA binding motif. Combined with work on PhoP glycosylation leading to altered DNA affinity, this work reaffirms the phenomenon of T3SS effector glycosyltransferases altering transcription factors to modulate gene expression. In addition to identifying additional glycosyltransferase targets, understanding the biochemical fundamentals of how glycosylation of different transcription factors can alter their DNA binding capacity and how it could differ among transcription factors merits additional research.

## Materials and methods

### Plasmids, strains, and cloning

The plasmids and strains used in this study are listed in Tables 1 and 2, respectively. Wild type *sseK1* (*Salmonella enterica*) and its derivative H244A E255A N256A, were cloned into pET42a using ABC cloning^66^. *ompR* and *qseF* were cloned in pTac using ABC cloning^66^. *ompR* and *sseK1/ompR* deletions were constructed using lambda red recombination with the pKD3 and pKD119 plasmids^67^. Mutants were screened on LB medium supplemented with 10 µg/mL chloramphenicol and mutations were confirmed by PCR and DNA sequencing. Protein purification was performed as described previously^14^. For the purification of glycosylated OmpR, His-tagged OmpR was co-expressed (or not) with FLAG-tagged SseK1 and purified against the His-epitope, as described previously^15^.

**Table 1:** Plasmids used in this study.

Construct	Plasmid	Source
Flag-SseK1	pFLAG-CTC-*sseK1*	^74^
GST-SseK1	pET42a-*sseK1*	^7^
GST-SseK1 (HEN)	pET42a-*sseK1* H244A E255A N256A	^74^
His-OmpR	pTac-*ompR*	This study
His-OmpR R15A	pTac-*ompR* R15A	This study
His-OmpR R122A	pTac-*ompR* R122A	This study
His-OmpR R15,122A	pTac-*ompR* R15,122A	This study
*ompF::mrfp*	pHG156a-*ompF::mrfp*	This study

**Table 2:** Strains used in this study.

Strain	Source
*S. Typhimurium* ATCC 1402 × pTac *ompR*	This study
*S. Typhimurium* ATCC 14028 × pTac *ompR* R15A	This study
*S. Typhimurium* ATCC 14028 × pTac *ompR* R122A	This study
*S. Typhimurium* ATCC 14028 × pTac *ompR* R15,122A	This study
*S. Typhimurium* ATCC 14028 × pTac* qseF*	This study
*S. Typhimurium ΔsseK1* × pTac *ompR*	This study
*S. Typhimurium ΔsseK2* × pTac* ompR*	This study
*S. Typhimurium ΔsseK3* × pTac* ompR*	This study
*S. Typhimurium ΔsseK1/sseK2* × pTac* ompR*	This study
*S. Typhimurium ΔsseK1/sseK3* × pTac* ompR*	This study
*S. Typhimurium ΔsseK2/sseK3* × pTac* ompR*	This study
*S. Typhimurium ΔsseK1/sseK2/sseK3* × pTac* ompR*	This study
*S. Typhimurium ΔompR*	This study
*S. Typhimurium ΔsseK1/ompR*	This study
*S. Typhimurium* × p*ompF* promoter::*mrfp*	This study
*S. Typhimurium ΔsseK1* × p*ompF* promoter::*mrfp*	This study
*S. Typhimurium ΔompR* × p*ompF* promoter::*mrfp*	This study
*S. Typhimurium ΔsseK1/ompR* × p*ompF* promoter::*mrfp*	This study
*S. Typhimurium ΔsseK1* × p*ompF* promoter::*mrfp* × *psseK1*	This study
*S. Typhimurium ΔsseK1* × p*ompF* promoter::*mrfp* × *pHEN*	This study
*S. Typhimurium ΔsseK1/ompR* × p*ompF* promoter::*mrfp* × *psseK1*	This study
*S. Typhimurium ΔsseK1/ompR* × p*ompF* promoter::*mrfp* × *pHEN*	This study
*Salmonella typhimurium ATCC 14028*	^75^
*S. typhimurium ΔsseK1*	^75^
*S. typhimurium ΔsseK2*	^75^
*S. typhimurium ΔsseK3*	^75^
*S. typhimurium ΔsseK1ΔsseK2*	^75^
*S. typhimurium ΔsseK1ΔsseK3*	^75^
*S. typhimurium ΔsseK2ΔsseK3*	^75^
*S. typhimurium ΔsseK1ΔsseK2ΔsseK3*	^75^

### Glycosyltransferase assay

In vitro glycosylation assays were conducted as previously described^7^. 200 nM SseK1 or SseK1 HEN were incubated in 50 mM Tris–HCl buffer pH 7.4, 1 mM UDP-GlcNAc, 10 mM MnCl_2_, and 1 mM DTT with 1 mM OmpR. After a 2-h incubation period at room temperature, samples were blotted with anti-R-GlcNAc and anti-His tag monoclonal antibodies (Abcam, Cambridge, MA, USA). Western blot images were captured in a LI-COR (LI-COR Biosciences, Lincoln, NY, USA) imager.

### Digest of gel-separated proteins

Affinity-purified proteins were separated by SDS-PAGE, fixed, and then visualized with Coomassie staining. Bands of interest were excised and Coomassie staining removed by destaining with 50 mM NH_4_HCO_3_, 50% ethanol for 20 min at room temperature with shaking at 750 rpm. Destained samples then dehydrated with 100% ethanol, before being reduced by being rehydrated with 10 mM DTT in 50 mM NH_4_HCO_3_. Samples were reduced for 1 h at 56 °C with shaking and then washed twice in 100% ethanol for 10 min to remove DTT. Reduced dehydrated gel bands were then rehydrated with 55 mM iodoacetamide in 50 mM NH_4_HCO_3_ and allowed to alkylate in the dark for 45 min at room temperature. Alkylation buffer was removed, and the gel samples washed with 50 mM NH_4_HCO_3_, followed by two rounds of 100% ethanol before being vacuum dried. Alkylated samples were then rehydrated with 20 ng/µL of trypsin (Promega) in 40 mM NH_4_HCO_3_ at 4 °C for 1 h. Excess protease was removed, gel pieces were covered in 40 mM NH_4_HCO_3_ and incubated overnight at 37 °C. Peptides were collected, desalted using homemade R3/C18 stage tips as previously described^68^ before analysis by LC–MS (Supplementary Fig. 1).

### Reverse phase LC–MS/MS

Peptide samples were resuspended in Buffer A* (2% MeCN, 0.1% TFA) and separated using a two-column chromatography set on a Dionex Ultimate 3000 UHPLC (Thermo Fisher Scientific). Samples were first concentrated on a PepMap100 C18 20 mm × 75 μm trap at 5 μl/min for 5 min with Buffer A (0.1% formic acid, 2% DMSO) and then separated on a PepMap C18 500 mm × 75 μm analytical column (Thermo Fisher Scientific). Separated peptide were infused into a Orbitrap Eclipse Mass Spectrometer (Thermo Fisher Scientific) at 300 nL/min for 65-min by altering the buffer composition from 2% Buffer B (0.1% formic acid, 77.9% acetonitrile, 2% DMSO) to 28% B over 35 min, then from 28% B to 4% B over 10 min, then from 40% B to 80% B over 5 min. The composition was held at 100% B for 5 min, and then dropped to 2% B over 1 min before being held at 2% B for another 9 min. The Eclipse Mass Spectrometer was operated in a data-dependent mode, acquiring one full precursor scan (resolution 120,000; 375–2000 m/z, AGC target of 1 × 10^6^) followed by up to 3 s of data-dependent HCD MS-MS events (using three collision energies of 25, 30, and 35; resolution 15k AGC target of 250% with a maximum injection time of 22 ms). 

### Mass spectrometry data analysis

Identification of Arg-glycosylation events was accomplished using MaxQuant (v1.6.17.0)^69^. The predicted amino acid sequences for OmpR were combined into a database with the *Escherichia coli* K12 proteome (Uniprot accession: UP000000625) the *Salmonella Typhimurium* SL1344 OmpR-his sequence and searched, allowing carbamidomethylation of cysteine set as a fixed modification and the variable modifications of oxidation of methionine and Arg-GlcNAcylation (H_13_C_8_NO_5_; 203.0793 Da to Arginine). Searches were performed with Trypsin cleavage specificity, allowing 2 miscleavage events with a maximum false discovery rate (FDR) of 1.0% set for protein and peptide identifications. The resulting modified peptide output was processed within the Perseus (v1.4.0.6)^70^ analysis environment to remove reverse matches and common protein contaminants. To ensure high quality data, assigned glycopeptides were manually assessed and the HCD spectra assigned to each unique glycopeptide annotated with the Interactive Peptide Spectral Annotator^71^ (http://www.interactivepeptidespectralannotator.com/PeptideAnnotator.html).

### mRFP reporter assay

A low-copy number plasmid (pHG165) carrying *ompF* promoter transcriptional fusions to mRFP (monomeric red fluorescent protein) was electroporated into *Salmonella*. 200 µL of LB media with Cb was used to grow the transformed bacteria in 96 well clear bottom black walled assay plates. mRFP expression levels were measured every 20 min of growth by a synergy H1 microplate reader. OD_600_ values were measured concurrently and mRFP data were presented as an average of RFU (Relative Fluorescence Units)/OD_600_ ratio.

### Electrophoretic mobility shift assay (EMSA)

A 5′ Alexa-fluor labelled DNA corresponding to the *Salmonella* *ompF* promoter region was amplified by PCR from *Salmonella *gDNA using the oligonucleotides: 5′ Alexa-fluor-tttttacgtcacactcaaggccagctatgctg-3′ and 5′-ttattaccctcattggtttttttatatgac-3′ as forward and reverse primers respectively. Two nmoles of purified PCR product were incubated for 10 min at room temperature in the presence of either OmpR or OmpR-GlcNAc in 10 μL buffer containing 50 mM HEPES, 100 mM K glutamate (pH 8.0), and 0.5 mg/mL BSA. Samples (10 µL) were loaded on 0.5% agarose gels and subjected to electrophoresis in 0.5 × TBE buffer. DNA–protein complexes were visualized by using a Li-COR Odyssey.

### Bile salt resistance and biofilm assays

Overnight cultures of *Salmonella* strains were diluted 1:100 to start a growth assay in LB with 0.6% or 0.3% Sodium deoxycholate in a 96 well plates with OD_600_ values measured every 3 h. For biofilm assays, overnight cultures of *Salmonella* strains were inoculated at 1:100 dilution into LB without sodium chloride into 96 well polystyrene plates. The plate was incubated at 30 °C without agitation. After 36 h of growth, the planktonic cells were removed, and wells were washed 3 times with PBS. Biofilm was fixed by adding 200 μL of methanol to the wells and incubating for 20 min at room temperature. 150 μL of 1% (w/v) crystal violet solution was added to the wells and incubated for 15 min. Wells were rinsed with PBS and air-dried. 150 μL of 30% (v/v) acetic acid was added to the wells and the plate was shaken gently to solubilize the crystal violet. The OD_570_ was measured to quantify biofilm.

## Supplementary Information

Supplementary Figure 1.

## Data Availability

The mass spectrometry proteomics data have been deposited to the ProteomeXchange Consortium via the PRIDE^72^ partner repository with the dataset identifier PXD039412.
