# Supplementary figures and images for: Salmonella T3SS effector SseK1 arginine-glycosylates the two-component response regulator OmpR to alter bile salt resistance
Source: Sci Rep. 2023 Jun 3;13:9018. doi: 10.1038/s41598-023-36057-9 (PMC10239501; doi:10.1038/s41598-023-36057-9)

Fig. 1A

R-GlcNac

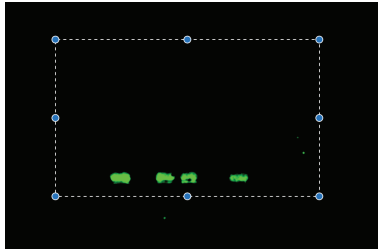

His

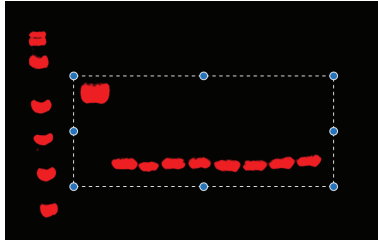

Merge

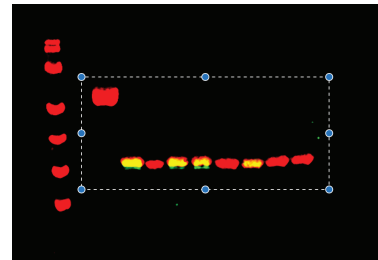

Fig. 1B

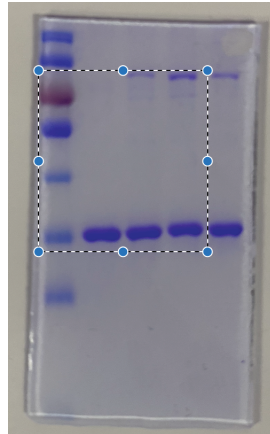

Fig. 1C

R-GlcNac

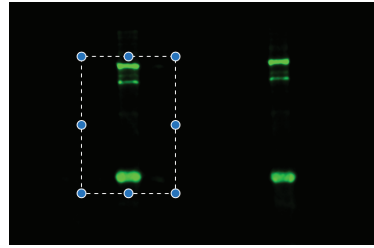

His

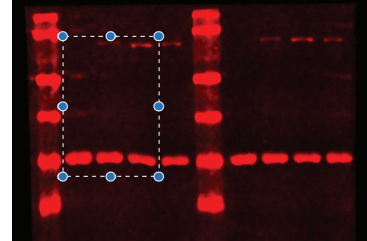

Merge

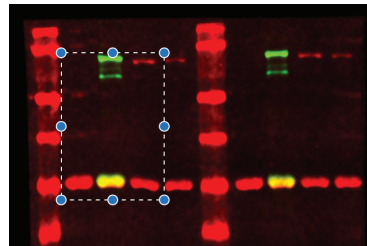

Fig. 2C

R-GlcNac

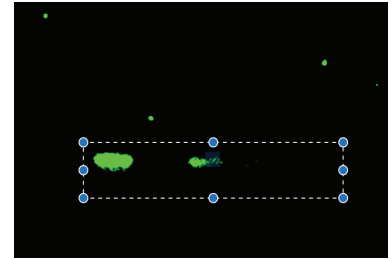

His

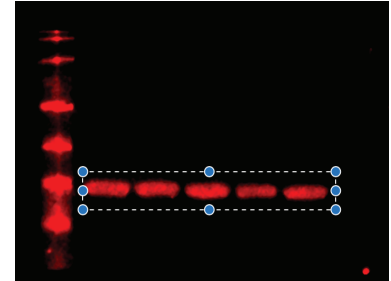

Merge

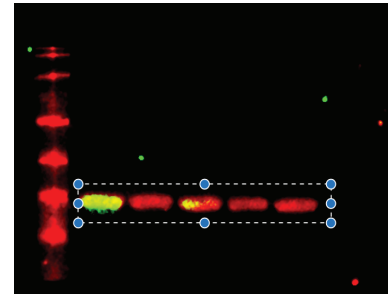

Fig 5A

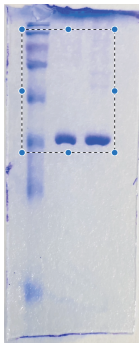

R-GlcNac

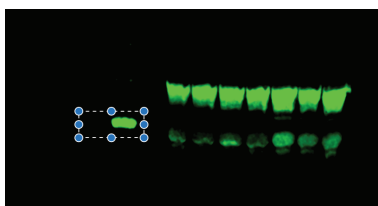

Supplement: Supplementary file 1 — Supplementary Figure 1. [file 41598_2023_36057_MOESM1_ESM.pdf]
